# Identification of novel compounds, oleanane- and ursane-type triterpene glycosides, from *Trevesia palmata*: their biocontrol activity against phytopathogenic fungi

**DOI:** 10.1038/s41598-018-32956-4

**Published:** 2018-09-28

**Authors:** Bora Kim, Jae Woo Han, Men Thi Ngo, Quang Le Dang, Jin-Cheol Kim, Hun Kim, Gyung Ja Choi

**Affiliations:** 1 Center for Eco-friendly New Materials, Korea Research Institute of Chemical Technology, Daejeon, 34114 Korea; 2 Department of Agricultural Chemistry, Chonnam National University, Gwangju, 61186 Korea; 3 Department of Pharmacology, Korea University of Science and Technology, Daejeon, 34113 Korea; 4 Research and Development Center of Bioactive Compounds, Vietnam Institute of Industrial Chemistry, Hanoi, Vietnam

## Abstract

Plants contain a number of bioactive compounds that exhibit antimicrobial activity, which can be recognized as an important source of agrochemicals for plant disease control. As part of our search for new antimicrobial agents from natural sources, we found that a crude methanol extract of *Trevesia palmata* exhibited a promising antifungal activity against phytopathogenic fungi, such as *Magnaporthe oryzae* and *Botrytis cinerea*. Furthermore, based on activity-guided fractionation, we isolated five antifungal compounds from the methanol extract of *T. palmata*: two new triterpene glycosides (TPGs), TPG1 (hederagenin-3-*O*-*β*-_D_-glucopyranosyl-(1  → 3)-*α*-_L_-rhamnopyranosyl-(1 → 2)-*α*-_L_-rhamnopyranosyl-(1 → 2)-*α*-_L_-arabinopyranoside) and TPG5 (3-*O*-*α*-_L_-rhamnopyranosyl asiatic acid), along with three known TPGs (TPG2 [macranthoside A], TPG3 [*α*-hederin], and TPG4 [ilekudinoside D]). The chemical structures of the TPGs were determined by spectroscopic analyses and by comparison with literature data. An *in vitro* antifungal bioassay revealed that except for TPG4 (ilekudinoside D; IC_50_ >256 μg/ml), the other TPGs exhibited strong antifungal activities against the rice blast pathogen *M. oryzae* with IC_50_ values ranging from 2–5 μg/ml. In particular, when the plants were treated with compound TPG1 (500 μg/ml), disease control values against rice blast, tomato grey mold, tomato late blight, and wheat leaf rust were 84, 82, 88, and 70%, respectively, compared to the non-treatment control. Considering the *in vitro* and *in vivo* antifungal activities of the TPGs and the *T. palmata* methanol extracts, our results suggest that *T. palmata* can be a useful source to develop new natural fungicides.

## Introduction

Plant pathogenic fungi threaten plant health and cause severe economic losses in crops and vegetable production^1^. Currently, synthetic fungicides are the primary means of controlling fungal diseases in the field and greenhouses. While the use of synthetic fungicides benefits agriculture by increasing productivity, their use has also led to concerns about environmental contamination, which is a potential risk to animal and human health^2^. Moreover, with the continued use of fungicides of the same class, several fungicides have become less effective due to the occurrence of resistant fungal pathogens^3^. Therefore, biological control using plant extracts or microbial antagonists has been considered as an alternative method for the control of plant diseases^4^.

Some of the diverse bioactive compounds contained in plants exhibit antimicrobial activities and can be used directly as pesticides due to their biodegradability and low toxicity^5^. Furthermore, substances isolated from plants are currently being used as lead compounds for synthetic or semisynthetic fungicides^6^. Extracts and phytochemicals from many herbs, spices, and medicinal plants have been explored for their effects on various plant diseases. For example, the bark extract and essential oil of *Drimys winteri* showed an antifungal activity *in vitro* and *in planta* against *Gaeumannomyce*s *graminis* var. *tritici*^7^. Chrysophanol, parietin, and nepodin extracted from *Rumex crispus* were effective for controlling barely and cucumber powdery mildews^8^. The extract of devil’s trumpet and metel (*Datura metel*) induced a systemic resistance in pearl millet (*Pennisetum glaucum*) against downy mildew^9^. Currently, several plant extracts and essential oils have been registered and sold as natural fungicides in the US and EU markets, including giant knotweed (*Reynoutria sachalinensis*) extract, thyme (*Thymus vulgaris*) oil, rosemary (*Rosemarinus officinalis*) oil, and tea tree (*Melaleuca alternifolia*) oil^10^.

Saponins are triterpenoidal or steroidal glycosides, widely distributed in a variety of plants^11^. Glycosides contain single or multiple sugars attached to one or more points of the basic skeleton. Accordingly, saponins have amphiphilic properties that interact with both lipophilic and hydrophilic components, which often have cytotoxic and other biological activities including antimicrobial activities^12,13^. The major mechanism of the antifungal activity of saponins is due to their ability to complex with sterols in fungal membranes and to cause a loss of the membrane integrity with the formation of transmembrane pores^14^. Indeed, triterpenoid saponins from the stem bark of *Polyscias fulva* have been reported to inhibit the growth of a wide range of yeasts and filamentous fungi^15^.

In the search for antifungal agents from Vietnamese plants, we found that a methanol extract of *Trevesia palmata* (Roxb. ex Lindl.) Vis. has the potential to control plant diseases caused by fungi. *T. palmata* in the family *Aralaceae* is a rainforest shrub with a canopy of intricate cut foliage that resembles giant snowflakes, which is distributed throughout India, Southeast Asia, and South America^16,17^. From this plant species, mono- and bisdesmosidic triterpene glycosides such as macranthoside A and hederacoside C were isolated, and their activity was investigated against human cancer cell lines^17^. Although thrombolytic, antiarthritic, antihyperglycemic, and analgesic activities have been reported from the leaf extracts of *T*. *palmata*, antimicrobial activities of *T. palmata* have not been investigated so far^17,18^. Therefore, the aim of this study was to further isolate and identify antifungal compounds from *T. palmata* and to explore the disease control efficacy of the *T. palmata* extract and the identified compounds. Consequently, we identified two new and three known compounds from *T. palmata*, and some of the isolated compounds exhibited *in vivo* and *in vitro* antifungal activities. Our results will provide useful information for development of plant-protecting agents using plant-derived materials to control plant diseases caused by phytopathogenic fungi.

## Results

### Suppression of plant diseases by *T. palmata* extracts

To find plant extracts showing a plant disease control activity, we have examined the *in vivo* antifungal activities of the methanol extracts of over 1,000 plant materials against seven fungal diseases: rice blast (RCB), rice sheath blight (RSB), tomato gray mold (TGM), tomato late blight (TLB), wheat leaf rust (WLR), barley powdery mildew (BPM), and pepper anthracnose (PAN). As shown in Table 1, the methanol extract (2,000 μg/ml) of *T*. *palmata* showed a disease control efficacy against RCB, TGM, and TLB with control values of 60, 79, and 43%, respectively, but there was no effect on RSB and BPM. In order to investigate the *in vivo* antifungal activity of the fractions obtained during the isolation of the active compounds from *T. palmata*, plants were treated with three organic fractions (*n*-hexane, ethyl acetate, and *n*-butanol) and one aqueous fraction prior to inoculation with the fungal pathogens. The results show that the ethyl acetate and *n*-butanol layers exhibited strong disease control efficacies without phytotoxicity, which was comparable to that of the methanol extract; however, the treatment of the *n*-hexane and water layers did not exhibit antifungal activities (Table 1). The ethyl acetate layer showed 70, 86, 57, and 50% for the control values against RCB, TGM, TLB, and PAN, respectively. The *n*-butanol layer showed 60, 75, 64, and 53% for the control values against RCB, TGM, TLB, and WLR, respectively. In contrast, three organic fractions and one aqueous fraction did not affect the disease development of RSB and BPM (Table 1). Thus, our results indicate that the ethyl acetate and *n*-butanol layers contain the active compounds exhibiting the antifungal activity against the fungal pathogens causing RCB, TGM, TLB, and WLR.

**Table 1:** Plant disease control efficacy of the *Trevesia palmata* methanol extract and its partitioned fractions.

Treatment	Disease control efficacy (%)						
	RCB	RSB	TGM	TLB	WLR	BPM	PAN
Methanol extract	60 ± 0^*^	0	79 ± 0^**^	43 ± 0^*^	3 ± 4	0	25 ± 11
*n*-Hexane layer	30 ± 14	0	7 ± 10	7 ± 10	13 ± 10	0	58 ± 12^*^
Ethyl acetate layer	70 ± 0^*^	5 ± 7	86 ± 0^**^	57 ± 0^**^	3 ± 4	0	50 ± 0^*^
*n*-Butanol layer	60 ± 14^*^	0	75 ± 6^**^	64 ± 10^**^	53 ± 0^**^	0	8 ± 11
Water layer	0	0	0	0	0	0	8 ± 11
Positive control	100^**^	100^**^	100^**^	100^**^	100^**^	100^**^	86 ± 0^**^

Methanol extract and each fraction (2,000 μg/ml) were applied onto plants one day prior to inoculation with fungal pathogens. As a positive control of each plant disease, blasticidin S (50 μg/ml) for RCB, validamycin (50 μg/ml) for RSB, fludioxonil (50 μg/ml) for TGM, dimethomorph (10 μg/ml) for TLB, flusilazole (10 μg/ml) for WLR, flusilazole (10 μg/ml) for BPM, and dithianon (50 μg/ml) for PAN were used. Control value (%) represent the mean ± standard deviation of two runs with three replicates. Asterisks indicate significant difference compared with negative controls by Tukey’s test (**p < 0.001, *p < 0.01). RCB, rice blast (caused by *Magnaporthe oryzae*); RSB, rice sheath blight (caused by *Rhizoctonia solani*); TGM, tomato gray mold (caused by *Botrytis cinerea*); TLB, tomato late blight (caused by *Phytophthora infestans*); WLR, wheat leaf rust (caused by *Puccinia triticina*); BPM, barley powdery mildew (caused by *Blumeria graminis* f. sp. *hordei*); and PAN, pepper anthracnose (caused by *Colletotrichum coccodes*).

### Structure determination of the oleanane-type glycosides

**Figure 1:**
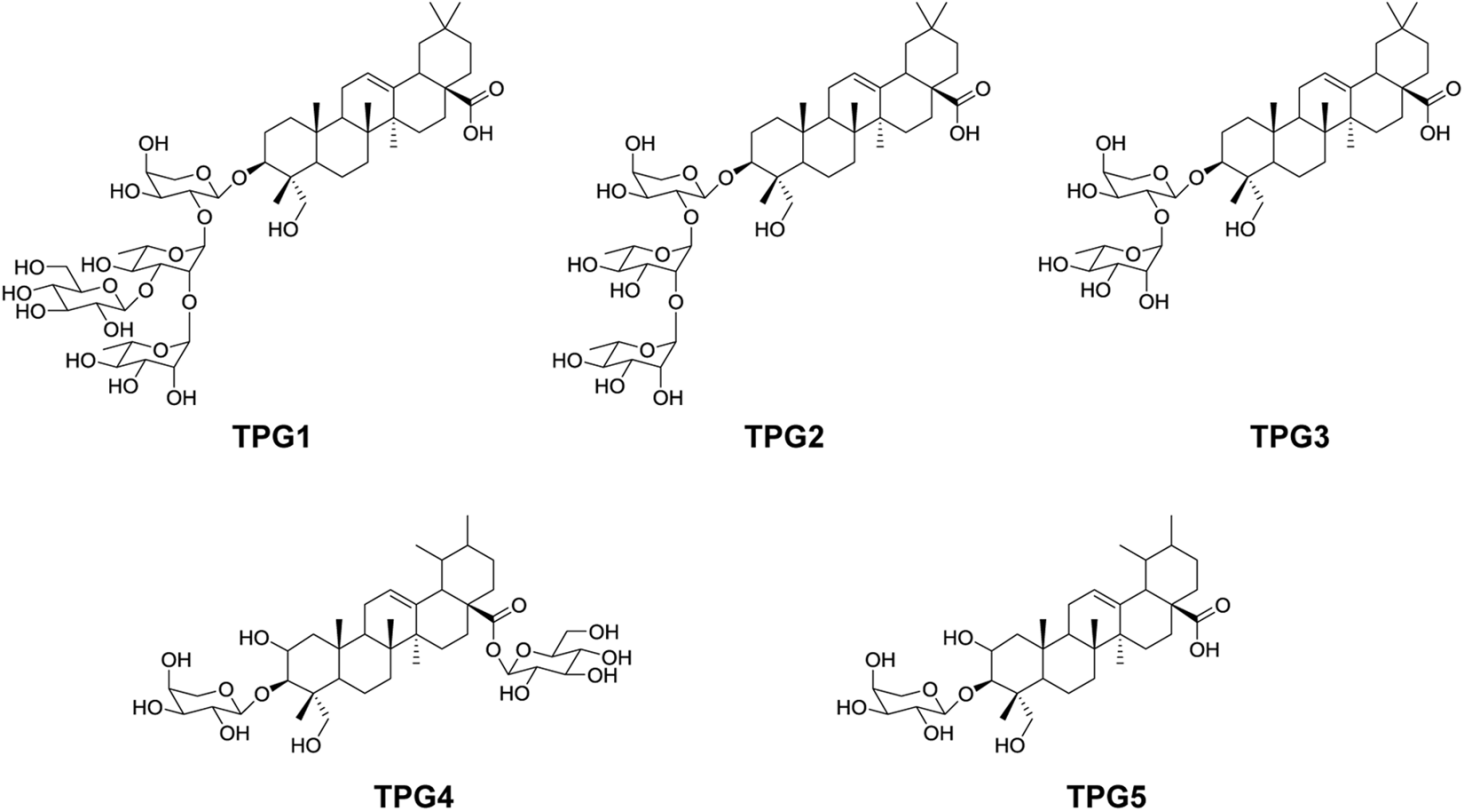
Chemical structures of the oleanane-type triterpene glycosides (TPG1, TPG2 [macranthoside A], and TPG3 [α-hederin]) and ursane-type triterpene glycosides (TPG4 [ilekudinoside D] and TPG5) isolated from *Trevesia palmata*.

**Figure 2:**
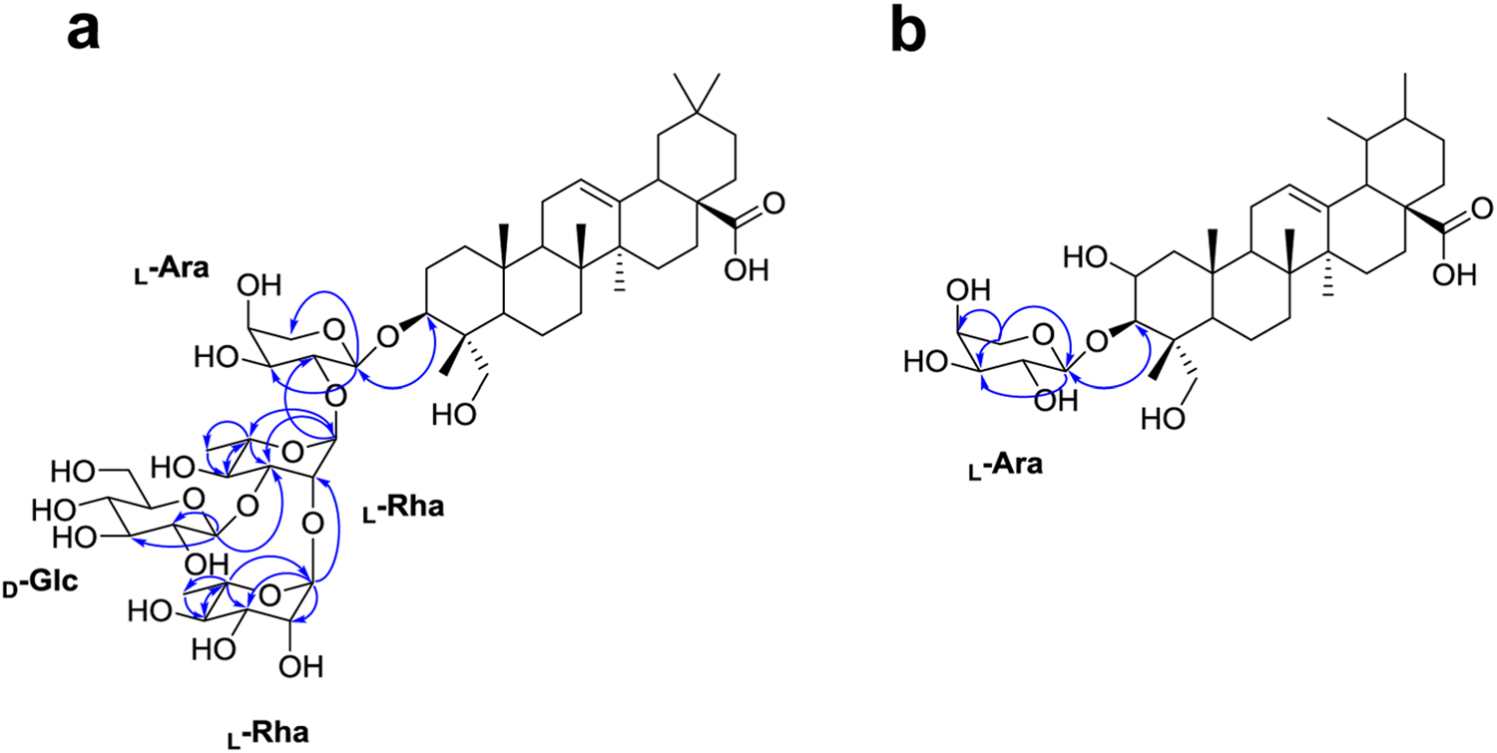
Key correlations to determine the positions of the glycosidic linkages in the oligosaccharide moiety of TPG1 (**a**) and TPG5 (**b**) in the HMBC spectra. The arrow lines represent the ^2^*J*(C,H) or ^3^*J*(C,H) correlation across the glycosidic linkages.

From the *T. palmata* extracts, we isolated and identified three oleanane-type triterpene glycosides (TPGs); compound TPG1 was newly identified in this study, and compounds TPG2 and TPG3 were identical as macranthoside A and *α*-hederin, respectively (Fig. 1). The molecular formula of TPG1 was determined as C_53_H_66_O_21_ from the [M + Na]^+^ ion at m/z 1081.5536 (Calc. m/z 1081.5559). Compound TPG1 showed significant fragment ion peaks at m/z 935 [M + Na − 146]^+^, m/z 773 [M + Na − 146–162]^+^ and m/z 627 [M + Na − 146 − 162 − 146]^+^ in the MALDIMS/MS, corresponding to the loss of two pentose units and one hexose unit (Supplementary Fig. S1). Acid methanolysis of TPG1 gave glucose, rhamnose, and arabinose in a 1:2:1 ratio (Supplementary Fig. S2). Compound TPG1 was obtained as an amorphous powder with [α]_D_^20^ −8.26. The IR spectrum showed absorption bands for the hydroxy (3378 cm^−1^), carbonyl (1692 cm^−1^), alkene (1636 cm^−1^) and methyl (1056 cm^−1^) groups in the molecule (Supplementary Fig. S3). The ^13^C-NMR spectrum showed 53 carbon signals, of which 30 were assigned to the aglycon moiety and 23 to a carbohydrate portion made up of four carbohydrate moieties (Tables 2 and 3). As shown in Table 2, the ^1^H-NMR spectrum of TPG1 displayed signals corresponding to six quaternary methyl groups (*δ*_H_ 0.70, 0.81, 0.91, 0.94, 0.97, and 1.18; H24-30), an olefinic proton (*δ*_H_ 5.24; H-12), an oxygen-bearing methine proton (*δ*_H_ 3.63; H-3), and a primary alcoholic function (*δ*_H_ 3.58 and 3.33; H-23). The ^13^C-NMR spectrum of TPG1 displayed signals for the methyl groups (*δ*_C_ 13.7, 16.4, 17.7, 26.5, 33.6, and 24.0), two olefinic carbons (*δ*_C_ 123.5 and 145.2), an oxygen-bearing carbon (*δ*_C_ 82.3), a primary alcoholic carbon (*δ*_C_ 64.4), and a carbonyl carbon (*δ*_H_ 182.0) (Table 2). Therefore, the spectroscopic data of the aglycon moiety of TPG1 were in complete agreement with those described previously for hederagenin^19^. The ^1^H-NMR spectrum of TPG1 showing the carbohydrate region corresponded to four anomeric protons at *δ*_H_ 5.19 (d, *J* = 1.8 Hz), 4.85 (d, *J* = 1.7 Hz), 4.52 (d, *J* = 7.8 Hz), and 4.47 (m), which gave correlations in the HMQC spectrum, with four anomeric carbon signals at *δ*_C_ 101.6, 102.8, 105.7 and 105.2, respectively (Table 3). Based on the HPLC analysis of the sugar moiety, the absolute configurations of the carbohydrate moieties for TPG1 were clearly identified (Supplementary Fig. S2). An exact determination of the sequence and linkages between the carbohydrate moieties was obtained from the HMBC spectrum, which showed key correlation peaks between the protons and carbons at *δ*_H_ 4.47 (*α*-_L_-Ara) and *δ*_C_ 82.3 (C-3 of aglycon), *δ*_H_ 5.19 (*α*-_L_-Rha I) and *δ*_C_ 76.7 (C-2 of *α*-_L_-Ara), *δ*_H_ 4.52 (*β*-_D_-Glc) and *δ*_C_ 82.9 (C-3 of *α*-_L_-Rha I), and *δ*_H_ 4.85 (*α*- _L_-Rha II) and *δ*_C_ 70.9 (C-2 of *α*-_L_-Rha I), indicating the connection of the aglycon to the sugar and the sugar to the sugar (Fig. 2a). Accordingly, the structure of compound TPG1 was proposed, in this study, as hederagenin-3-*O*-*β*-_D_-glucopyranosyl-(1 → 3)-*α*-_L_-rhamnopyranosyl-(1 → 2)-*α*-_L_-rhamnopyranosyl-(1 → 2)-*α*-_L_-arabinopyranoside (Fig. 1).

**Table 2:** NMR spectroscopic data for the aglycon moieties of the triterpene glycosides (recorded in methanol-*d*_4_; *δ* in ppm; *J* in Hz). Assignments aided by HMQC and HMBC experiments. *mult*., multiplicity.

Position	TPG1		TPG2		TPG3		TPG4		TPG5	
	*δ*_C_	*δ*_H_ *mult*. (*J*)	*δ*_C_	*δ*_H_ *mult*. (*J*)	*δ*_C_	*δ*_H_ *mult*. (*J*)	*δ*_C_	*δ*_H_ *mult*. (*J*)	*δ*_C_	*δ*_H_ *mult*. (*J*)
1	39.6	1.67-1.57 *m*; 0.98 *m*	39.7	1.70-1.58 *m*; 0.98 *m*	39.7	1.70-1.58 *m*; 0.98 *m*	47.4	2.05 *dd* (12.6, 4.6); 0.88 *m*	47.4	2.04 *m*; 0.88 *m*
2	26.6	1.93-1.85 *m*; 1.81-1.68 m	26.6	1.95-1.85 *m*; 1.80-1.71 *m*	26.5	1.95-1.85 *m*; 1.80-1.71 *m*	68.0	3.82 *dd* (12.0, 2.0)	68.0	3.80 *m*
3	82.3	3.63 *dd* (10.2, 3.6)	82.4	3.69 *dd* (11.8, 5.0)	82.3	3.62 *dd* (11.8, 4.7)	88.3	3.48 *d* (9.5)	88.3	3.48 *dd* (9.5, 2.4)
4	43.9		44.0		43.9		45.2		45.2	
5	48.0	1.30-1.27 *m*	48.1	1.30-1.27 *m*	48.1	1.30-1.27 *m*	47.7	1.33 *m*	47.8	1.33 *m*
6	18.8	1.49 *d* (12.3); 1.44-1.32 *m*	18.8	1.50 *d* (13.2); 1.42-1.33 *m*	18.8	1.50 *d* (12.3); 1.42-1.33 *m*	18.8	1.52 *m*; 1.40 *m*	18.8	1.52 *m*; 1.40 *m*
7	33.8	1.67-1.57 *m*; 1.30-1.27 *m*	33.8	1.70-1.58 m; 1.30-1.27 *m*	33.9	1.70-1.58 *m*; 1.30-1.27 *m*	33.6	1.68 *m*; 1.33 *m*	33.7	1.68 *m*; 1.33 *m*
8	40.4		40.5		40.5		41.0		40.8	
9	49.0	1.67-1.57 *m*	49.9	1.70-1.58 *m*	49.5	1.70-1.58 *m*	48.9	1.65 *m*	48.9	1.65 *m*
10	37.6		37.6		37.6		38.5		38.6	
11	24.5	1.93-1.85 *m*	24.5	1.92-1.85 *m*	24.5	1.92-1.85 *m*	24.5	2.0 *m*; 0.98 *m*	24.5	2.01 *m*; 0.98 *m*
12	123.5	5.24 *t* (3.7)	123.6	5.24 *t* (3.8)	123.6	5.24 *t* (3.7)	127.0	5.28 *t* (3.8)	126.4	5.26 *t* (3.6)
13	145.2		145.3		145.5		139.3		140.1	
14	42.9		43.0		43.0		43.4		43.0	
15	28.8	1.81-1.68, *m*; 1.08, *m*	28.9	1.80-1.71 *m*; 1.08 *dt* (13.6, 3.5)	28.9	1.80-1.71 *m*; 1.08 *dt* (13.6, 3.5)	29.2	1.96 *m*; 1.12 *m*	29.3	1.96 *m*; 1.12 *m*
16	24.0	2.01 *td* (13.4, 3.9); 1.67-1.57 *m*	24.0	2.00 *td* (13.6, 4.1); 1.70-1.58 *m*	24.1	1.99 *td* (13.6, 3.9); 1.70-1.58 *m*	25.2	2.10 *m*; 1.78 *m*	25.4	2.10 *m*; 1.78 *m*
17	47.6		47.7		47.8		49.4		49.9	
18	42.7	2.85 *dd* (13.9, 4.5)	42.8	2.85 *dd* (13.8, 4.5)	43.9	2.86 *dd* (13.9, 4.5)	54.2	2.26 *d* (11.3)	54.5	2.25 *d* (11.5)
19	47.2	1.81-1.68 *m*; 1.13 *m*	47.3	1.80-1.71 *m*; 1.13 *ddd* (13.7, 4.7, 2.3)	47.4	1.80-1.71 *m*; 1.13 *ddd* (13.7, 4.7, 2.3)	40.4	1.40 *m*	40.5	1.40 *m*
20	31.6		31.6		31.6		40.2	0.93 *m*	40.2	0.93 *m*
21	34.9	1.44-1.32 *m*; 1.22 *m*	34.9	1.42-1.33 *m*; 1.20, *m*	35.0	1.42-1.33 *m*; 1.20 *m*	31.7	1.52 *m*; 1.33 *m*	31.7	1.52 *m*; 1.33 *m*
22	33.3	1.67-1.57 *m*; 1.54 *d* (13.6)	33.4	1.70-1.58 *m*; 1.54 *dt* (13.7, 3.6)	33.4	1.70-1.58 *m*; 1.53 *dt* (14.5, 3.7)	37.5	1.78 *m*; 1.65 *m*	37.6	1.78 *m*; 1.65 *m*
23	64.4	3.58 *m*; 3.33 *m*	64.5	3.62 *dd* (12.0, 4.6); 3.33 *d* (8.2)	64.6	3.62 *m*; 3.33 *m*	64.0	3.71 *m*; 3.29 *d* (11.4)	64.0	3.71 *m*; 3.29 *d* (11.4)
24	13.7	0.70 *s*	13.7	0.70 *s*	13.7	0.70 *s*	14.4	0.77 *s*	14.4	0.77 *s*
25	16.4	0.97 *s*	16.4	0.97 *s*	16.4	0.98 *s*	17.9	1.08 *s*	17.9	1.08 *s*
26	17.7	0.81 *s*	17.8	0.82 *s*	17.9	0.83 *s*	18.0	0.86* s*	18.0	0.86 *s*
27	26.5	1.18 *s*	26.5	1.18 *s*	26.5	1.18 *s*	24.1	1.15 *s*	24.1	1.16 *s*
28	182.0		182.0		182.6		177.9		182.8	
29	33.6	0.91 *s*	33.6	0.91 *s*	33.6	0.90 *s*	17.6	0.92 *d* (6.4)	17.6	0.91 *d* (6.3)
30	24.0	0.94 *s*	24.1	0.94 *s*	24.0	0.94 *s*	21.6	0.99 *s*	21.7	0.99 *s*

**Table 3:** NMR spectroscopic data for the oligosaccharide moieties of the triterpene glycosides (recorded in methanol-*d*_4_; *δ* in ppm; *J* in Hz). Assignments aided by HMQC and HMBC experiments. *mult*., multiplicity.

Position	TPG1		TPG2		TPG3		TPG4		TPG5	
	*δ*_C_	*δ*_H_ *mult*. (*J*)^c^	*δ*_C_	*δ*_H_ *mult*. (*J*)	*δ*_C_	*δ*_H_ *mult*. (*J*)	*δ*_C_	*δ*_H_ *mult*. (*J*)^b^	*δ*_C_	*δ*_H_ *mult*. (*J*)^b^
*α*-_L_-Ara (C-3)										
1	105.2,	4.47 *m*	105.0	4.49 *dd* (5.0, 1.5)	104.4	4.56 *dd* (5.0, 1.5)	106.3	4.31 *d* (7.6)	106.3	4.31 *d* (7.6)
2	76.7	3.64 *m*	76.9	3.65 *m*	76.9	3.71 *m*	72.9	3.71 *m*	72.9	3.72 *d* (11.5)
3	74.1	3.64 *m*	74.0	3.65 *m*	74.0	3.71 *m*	74.6	3.54 *m*	74.6	3.53 *dd* (9.6, 3.4)
4	69.9	3.76 *m*	69.8	3.76 *m*	69.8	3.77 *m*	70.1	3.85 *dt* (3.5, 1.6)	70.1	3.84 *dt* (3.3, 1.5)
5	66.0	3.84 *m*;3.52 *dd* (12.4, 1.9)	65.7	3.84 *dd* (12.3, 3.9); 3.51 *dd* (12.3, 2.2)	65.7	3.84 *dd* (12.0, 5.0); 3.50 *dd* (11.6, 2.2)	67.8	3.93 *dd* (12.7, 2.1); 3.65 *dd* (12.8, 1.3)	67.8	3.93 *dd* (12.7, 2.1); 3.65 *d* (12.4)
*α*-_L_-Rha										
1	101.6	5.19 *d* (1.8)	101.7	5.17 *d* (1.9)	101.9	5.16 *d* (1.7)				
2	70.9	4.25 *dd* (3.2, 1.8)	71.1	4.24 *dd* (3.2, 1.9)	71.1	3.91 *dd* (3.5, 1.7)				
3	82.9	3.87 *dd* (9.5, 3.1)	82.8	3.88 *dd* (9.5, 3.0)	82.8	3.48 *d* (11.2)				
4	72.4	3.56 *m*	72.3	3.56 *t* (9.5)	72.3	3.37 *q* (9.5)				
5	70.0	3.93 *dq* (9.6, 6.2)	70.1	3.92 *dq* (9.7, 6.2)	70.1	3.85 *q* (6.2)				
6	17.8	1.27 *d* (6.0)	18.1	1.26 *d* (6.2)	18.1	1.24 *d* (6.3)				
*β*-_D_-Glc										
1	105.7	4.52 *d* (7.8)	105.8	4.51 *d* (7.8)						
2	75.44	3.36 *m*	75.3	3.31 *dd* (7.9, 1.32)						
3	76.6	3.39 *m*	77.8	3.38 *t* (8.9)						
4	73.7	3.42 *d* (9.5)	71.3	3.33 *d* (11.2)						
5	76.3	3.48 *t* (9.0)	77.8	3.57 *d* (11.3)						
6	61.6	3.82 *m*; 3.67 *dd* (12.0, 3.7)	62.4	3.87 *dd* (11.9, 2.4); 3.86 *dd* (12.3, 3.9)						
*α*-_L_-Rha (terminal)										
1	102.8	4.85 *d* (1.7)								
2	72.1	3.64 *m*								
3	79.0	3.59 *m*								
4	72.39	3.84 *m*								
5	70.6	3.99 *dq* (9.5, 6.2)								
6	18.2	1.26 *d* (6.0)								
*β*-_D_-Glc (C-28)										
1							95.7	5.37 *d* (8.1)		
2							73.9	3.33 *m*		
3							78.2	3.35 *d* (8.5)		
4							71.1	3.39 *m*		
5							78.5	3.42 *m*		
6							62.5	3.80 *m*; 3.71 *m*

The ^1^H- and ^13^C-NMR spectral data of compounds TPG2 and TPG3 were fully superimposable to the spectral data of TPG1, except for the signals of the sugar chain attached at C-3 of the aglycon moiety, indicating that TPG2 and TPG3 possessed hederagenin as the aglycon (Tables 2 and 3, and Supplementary Fig. S4). Compound TPG2 showed a quasi-molecular ion peak [M + Na]^+^ at m/z 935 in the MALDI-MS, and significant fragment ion peaks at m/z 773 [M + Na − 162]^+^ and 627 [M + Na − 162 − 146]^+^ in the MALDIMS/MS, corresponding to the loss of one pentose unit and one hexose unit (Supplementary Fig. S1). NMR data of compound TPG2 with comparison of TPG1 showed that TPG2 differs from TPG1 only by the absence of the rhamnopyranosyl unit linked at C-2 of rhamnose I (Table 3 and Supplementary Fig. S4). Therefore, we concluded that compound TPG2 is identical as macranthoside A, which is hederagenin 3-*O*-*β*-_D_-glucopyranosyl-(1 → 3)-*α*-_L_-rhamnopyranosyl-(1 → 2)-*α*-_L_-arabinopyranoside (Fig. 1)^20,21^.

Compound TPG3 showed a quasi-molecular ion peak [M + Na]^+^ at m/z 773 in the MALDI-MS, and significant fragment ion peak at m/z 627 [M + Na − 146]^+^ in the MALDIMS/MS, corresponding to the loss of one pentose unit (Supplementary Fig. S1). Analysis of the NMR data of compound TPG3 based on a comparison with TPG2 showed that TPG3 differs from TPG2 only by the absence of the glucopyranosyl unit linked at C-2 of rhamnose I (Table 3 and Supplementary Fig. S4). Therefore, the compound TPG3 was identical as *α*-hederin, which is hederagenin 3-*O*-*α*-_L_-rhamnopyranosyl-(1 → 2)-*α*-_L_-arabinopyranoside (Fig. 1)^20,21^.

### Structure determination of the ursane-type of glycosides

Two ursane-type TPGs were identified in this study from the *T. palmata* extracts; compound TPG4 was identical to ilekudinoside C, and TPG5 was a previously unreported compound (Fig. 1). Compound TPG4 showed a quasi-molecular ion peak [M + Na] ^+^ at m/z 805 in the ESI-MS. The ^13^C-NMR spectrum of TPG4 showed 41 carbon signals, of which 30 were assigned to the aglycon moiety and 11 to a carbohydrate portion made up of two carbohydrate moieties (Tables 2 and 3). The NMR spectroscopic data of TPG4 displayed the proton signals for six methyls (*δ*_H_ 0.77, 0.86, 0.92, 0.99, 1.08, and 1.15; H24–30), an olefin (*δ*_H_ 5.28; H-12), two oxygen-bearing methines (*δ*_H_ 3.82 and 3.48; H-2 and H-3), and a primary alcohol (*δ*_H_ 3.71 and 3.29; H-23), along with six carbon signals for the methyls (*δ*_C_ 14.4, 17.6, 17.9, 18.0, 21.6, and 24.1), two olefins (*δ*_C_ 127.0 and 139.3), two oxygen-bearing methines (*δ*_C_ 68.0 and 88.3), a primary alcohol (*δ*_C_ 64.0), and a carbonyl (*δ*_H_ 177.9) (Table 2). Therefore, the spectroscopic data of TPG4 including the aglycon moiety were in complete agreement with those described previously for asiatic acid (Table 2)^22^. The ^1^H-NMR spectrum showing the carbohydrate region corresponded to two anomeric protons at *δ*_H_ 5.37 (d, *J* = 8.1 Hz) and 4.31 (d, *J* = 7.6 Hz) with two anomeric carbon signals at *δ*_C_ 106.3 and 95.7, respectively (Table 3). An exact determination of the linkages between the aglycon and carbohydrate moieties was obtained from the HMBC spectrum, which showed key correlation peaks between the protons and carbons at *δ*_H_ 4.31 (*α*-_L_-Ara) and *δ*_C_ 88.3 (C-3 of aglycon), and *δ*_H_ 5.37 (*β*-_D_-Glc) and *δ*_C_ 177.9 (C-28 of aglycon), indicating the connection of the aglycon to the sugar. Therefore, the compound TPG4 was identical to ilekudinoside C which is 3-*O*-*α*-_L_-arabinopyranosyl asiatic acid 28-*O*-*β*-_D_-glucopyranosyl ester (Fig. 1)^23^.

Compound TPG5 showed a quasi-molecular ion peak [M + Na]^+^ at m/z 643. Its molecular formula was determined to be C_35_H_56_O_9_ by HR-ESI-MS at m/z 643.5520 [M + Na]^+^ (Calc. m/z 643.3822 for C_35_H_56_O_9_Na). Based on the MS and NMR analyses, TPG5 differed from TPG4 only by the absence of the glucopyranosyl unit linked at C-28 of the aglycon (Fig. 1 and Table 3). The ^3^*J* correlation between *δ*_H_ 4.31 (*α*-_L_-Ara) and *δ*_C_ 88.3 (C-3 of aglycon) in the HMBC spectrum indicated the arabinose moiety linked at 3-*O*-position of the aglycon skeleton (Fig. 2b). Therefore, the structure of 3-*O*-*α*-_L_-arabinopyranosyl asiatic acid was assigned to TPG5 (Fig. 1), which has not been reported before.

### *In vitro* antifungal activities of the isolated compounds

Among the six fungal pathogens tested in this study, the causal agent *Magnaporthe oryzae* for rice blast disease was the most sensitive to the oleanane-type TPG1, TPG2, and TPG3 as well as the ursane-type TPG5 (Table 4). Moreover, the *in vitro* antifungal assay with crude extracts of *T. palmata* showed that the growth of *M. oryzae* was completely inhibited by the methanol, ethyl acetate, and *n*-butanol extracts at a concentration of 125 μg/ml (data not shown). The minimum inhibitory concentration (MIC) values for TPG1, TPG2, TPG3, and TPG5 were 8, 8, 4, and 16 μg/ml against *M. oryzae*, respectively, whereas the MIC values for all the other tested microorganisms were over 256 μg/ml (data not shown). All the TPGs except for TPG4 also had relatively low half maximal inhibitory concentration (IC_50_) values for *Botrytis cinerea* (for tomato gray mold) and *Phytophthora infestans* (for tomato late blight) as well as for *M. oryzae* (Table 4). Compound TPG1 exhibited an activity with IC_50_ values of 4, 34, and 136 μg/ml against *M. oryzae*, *B. cinerea* and *P. infestans*, respectively, which were comparable to those of TPG2, TPG3, and TPG5 (Table 4). In contrast, compound TPG4 did not exhibit any antifungal activity against all the tested fungal species.

**Table 4:** *In vitro* antifungal activity of the triterpene glycosides against plant pathogenic fungi.

Fungal pathogen	IC_50_ (μg/ml)				
	TPG1	TPG2	TPG3	TPG4	TPG5
*Alternaria porri*	>256	>256	>256	>256	>256
*Botrytis cinerea*	34 ± 7^*^	24 ± 11^*^	44 ± 17^*^	>256	35 ± 7^*^
*Colletotrichum coccodes*	>256	>256	>256	>256	>256
*Fusarium oxysporum*	>256	>256	>256	>256	>256
*Magnaporthe oryzae*	4 ± 0^*^	5 ± 1^*^	2 ± 0^*^	>256	5 ± 2^*^
*Phytophthora infestans*	136 ± 13^*^	205 ± 26	81 ± 33^*^	>256	128 ± 0^*^

IC_50_ values (μg/ml) represent the mean ± standard deviation of two runs with three replicates. Asterisks indicate significant difference compared with negative controls by Tukey’s test (*p* < 0.001).

### Effects of compound TPG1 on the development of plant diseases caused by fungi

**Figure 3:**
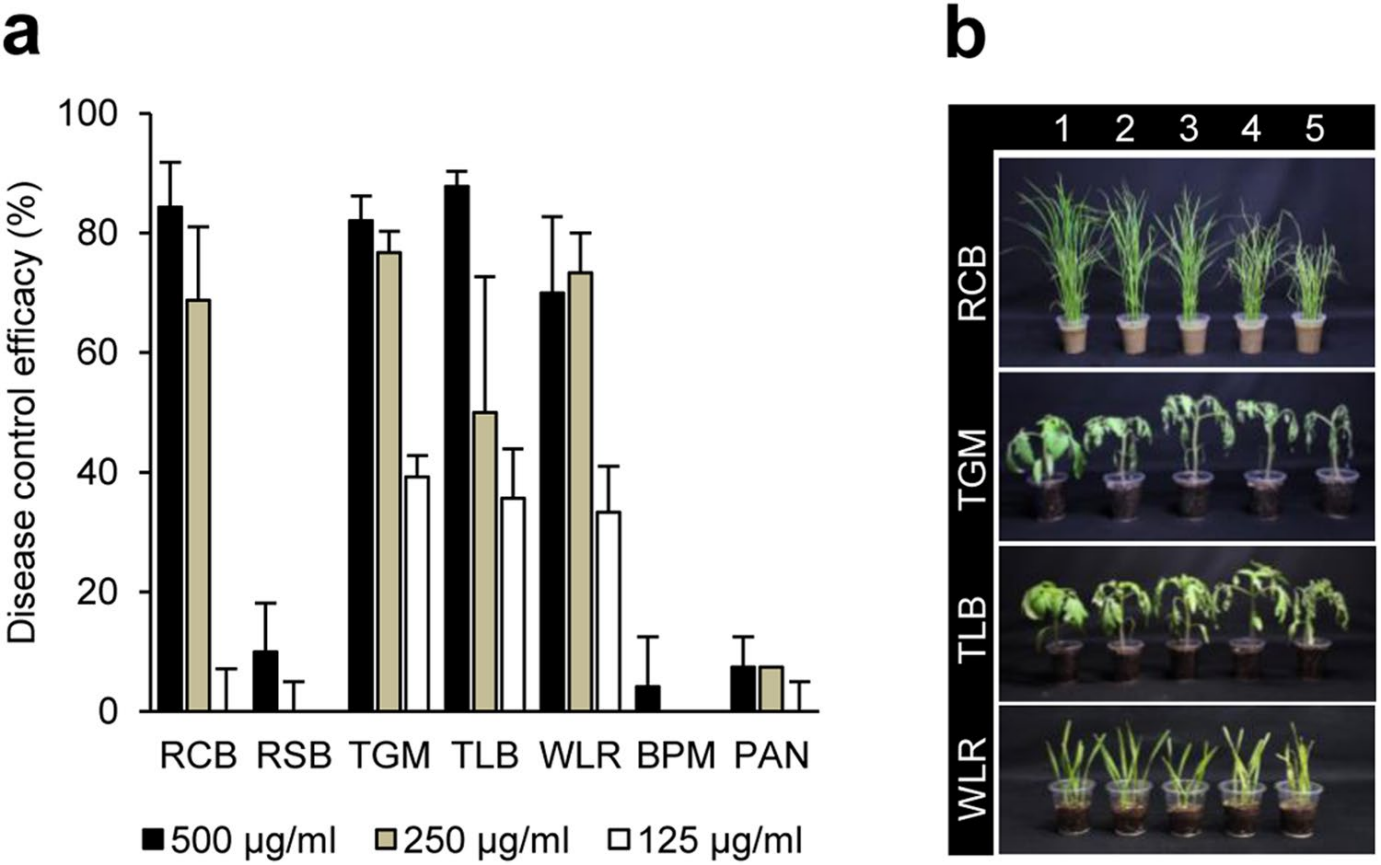
Effects of compound TPG1 on the development of plant diseases. (**a**) Control efficacy of TPG1 isolated from *Trevesia palmata* against seven plant diseases. RCB, rice blast; RSB, rice sheath blight; TGM, tomato gray mold; TLB, tomato late blight; WLR, wheat leaf rust; BPM, barley powdery mildew; PAN, pepper anthracnose. (**b**) Representatives of the plants treated by TPG1. Plants were inoculated with spores or mycelial suspensions of phytopathogenic fungi one day after treatment with TPG1. 1, treatment with a chemical fungicide as a positive control; 2–4, treatment with 500, 250, 125 μg/mL of TPG1, respectively; 5, treatment with the Tween 20 solution containing 5% methanol as a negative control. The bars represent the mean ± standard deviation of two runs with three replicates. Asterisks indicate significant difference compared with negative controls by Tukey’s test (**p < 0.001, *p < 0.01).

To investigate the *in vivo* antifungal activity of compound TPG1 isolated from *T. palmata*, plants were treated with 125, 250, and 500 μg/ml of TPG1 prior to inoculation with the fungal pathogens. Other TPGs were not available for the *in vivo* antifungal activity assay due to the limited quantities. The treatment with TPG1 (500 μg/ml) exhibited disease control values of 84, 82, 88, and 70%, respectively, against RCB, TGM, TLB, and WLR, whereas TPG1 had no effects of disease control on RSB, BPM, and PAN (Fig. 3). At a concentration level of 125 μg/ml, TPG1 exhibited disease control values that ranged from 35–40% against TGM, TLB, and WLR (Fig. 3). In addition to the disease control efficacy, we observed that no phytotoxic symptoms appeared on the TPG1-treated plants.

## Discussion

In this study, we found that the methanol extract of *T. palmata* exhibited *in vivo* and *in vitro* antifungal activities, and two previously unreported triterpene glycosides (TPG1 and TPG5) and three known triterpene glycosides (TPG2 [macranthoside A], TPG3 [*α*-hederin], and TPG4 [ilekudinoside D]) were identified from the methanol extract based on the spectroscopic analysis. Macranthoside A and *α*-hederin have previously been isolated from a variety of plants, such as Japanese angelica tree (*Aralia elata*) and ivy plants (*Hedera* spp.), and these compounds exhibited antimicrobial, antitumor, and cytoprotective activities^24–29^. Ilekudinoside D was first isolated from the leaves of *Ilex kudincha* in the course of screening for acyl CoA cholesteryl acyl transferase inhibitors, but its biological activity was not further characterized^23^.

Saponins are triterpenoidal or steroidal glycosides which are found in a variety of plants^30^. A new saponin, TPG1, was a major active constituent of the crude extract of *T. palmata* in the present study. Our results show that TPG1 can effectively control rice blast, tomato grey mold, tomato late blight, and wheat leaf rust without phytotoxic effects, which was consistent with previous observations that saponin molecules were effective for plant disease control^12,31,32^. The saponin mixtures from alfalfa prevented the fungal infection of *M. oryzae* on different rice cultivars^31^. Aalliospiroside A isolated from shallot (*Allium cepa*) was able to protect strawberry plants from the fungal pathogen *Colletotrichum gloeosporioides*^12^. Wilfosides and cynauricuoside A from the *Cynanchum wilfordii* root were effective for controlling barley powdery mildew caused by *Blumeria graminis* f. sp. *hordei*^32^. Additionally, most saponin compounds as well as TPG1 did not show any phytotoxic effects in various crops, suggesting their potential roles as a natural fungicide for the control of plant diseases^12,31,32^.

Compounds TPG4 and TPG5 are ursane-type triterpenoid glycosides containing asiatic acid as an aglycon backbone. Related with the antimicrobial activities, Shi *et al*.^33^ and Lahlou *et al*.^34^ described the potent antimicrobial property of asiatic acid. However, TPG4, which corresponds to ilekudinoside D, did not show any antifungal activity, although the new compound TPG5 (3-*O*-*α*-_L_-arabinopyranosyl asiatic acid) exhibited an inhibitory activity against plant pathogenic fungi (Table 4). From these observations, we hypothesized that in compound TPG4, the esterification of a C-28 carboxyl group with *β*-_D_-glucose may have a negative effect on the antimicrobial property. This hypothesis could be supported by the following previous investigations on the structure−activity relationship of saponins^31,35,36^. Bisdesmosidic saponins containing saccharide chains at both C-3 and C-28 positions, which were similar to TPG4, exhibited a lower antifungal activity compared to those of monodesmosidic saponins^31,35,36^. Macranthoside A and medicagenic acid 3-*O*-*β*-_D_-glucopyranoside (monodesmosidic compounds) isolated from alfalfa (*Medicago sativa*) exhibited more antifungal activity against *M. oryzae* compared to bisdesmosides^31^. Nudicaucins A–C and guaiacin D containing saccharide chains at both the C-3 and C-28 positions, which were isolated from *Hedyotis nudicaulis*, showed a very weak activity against *Bacillus subtilis*^36^. Furthermore, it has been reported that the sugar chain attached to C-3 of saponins is usually significant for both the membranolytic and antifungal activity for *Fusarium oxysporum* f. sp. *lycopersici*, *Septoria lycopersici* and *B. cinerea*, and the removal of these sugar residues often results in a loss of biological activity^13,37^. Therefore, we suggest that the absence of antifungal activities from TPG4 seems to be due to the esterification of a C-28 carboxyl group with *β*-_D_-glucose.

Our observations that the TPGs exhibit antifungal activities could be explained by the membranolytic action through which these compounds are able to interfere with the normal growth of plant pathogenic fungi. Saponins haven been known to form a complex with sterol or other membrane components which results in a change in the permeability or a disruption of the fungal cell membrane^38^. Therefore, the inhibitory effect of saponins may vary depending on the microorganisms. In present study, the inhibitory effect of saponins differed depending on the fungal species; the most sensitive fungus to the TPGs was *M. oryzae*, whereas *Alternaria porri*, *Colletotrichum coccodes*, and *F. oxysporum* were fully resistant to the TPGs (Table 4). In addition to the fungal pathogens, we investigated the antibacterial activities of the TPGs against seven plant pathogenic bacteria: *Agrobacterium tumefaciens*, *Burkholderia glumae*, *Clavibacter michiganesis* subsp. *michiganesis*, *Pectobacterium carotovorum* subsp. *carotovorum*, *Pseudomonas syringae* pv. *actinidiae*, *Ralstonia solanacearum*, and *Xantomonas arboricola* pv. *pruni*. Our results show that a Gram-positive bacterium *Clavibacter michiganensis* subsp. *michiganensis* was exclusively sensitive to the TPGs among all the tested bacterial species (Supplementary Table S1). The results also support the hypothesis that the inhibitory effect of saponins may vary depending on the microorganisms; Gram-positive bacteria comprised of a single layered cell wall are more sensitive to saponins than Gram-negative bacteria because saponin molecules are likely involved in the mechanical damage of Gram-positive bacteria cell membranes^39^.

In conclusion, we isolated and identified five triterpene glycosides from the methanol extract of *T. palmata*, and compound TPG1 was a major active constituent of the crude extract of *T. palmata*. Of the five glycoside compounds, the oleanane-type glycoside TPG1 and ursane-type glycoside TPG5 were identified as new compounds. Furthermore, *in vitro* and *in vivo* antifungal assays revealed that the TPGs except for TPG4 are effective in controlling the fungal pathogens *M. oryzae*, *B. cinerea*, *P. infestans*, and *Puccinia triticina* on each host. Our results suggest that *T. palmata* extract, which includes triterpene glycosides, could be useful for the development of natural fungicides as an alternative or supplement to current management practices for plant diseases.

## Methods

### Plant materials and fungal pathogens

The aerial parts of *T. palmata* were collected by the Department of Phytochemistry at the Vietnam Institute of Industrial Chemistry (Hanoi, Vietnam), and voucher specimens were deposited in the laboratory of Dr. Tran Bach at the Institute of Ecology and Biological Resources (Hanoi, Vietnam). The collected plant materials were air-dried and finely macerated for further study.

Phytopathogenic fungus *Alternaria porri, B. graminis* f. sp. *hordei, B. cinerea*, *C. coccodes*, *F. oxysporum*, *M. oryzae*, *P. infestans*, and *P. triticina* were used for the *in vitro* or *in vivo* antifungal activity assay. Fungal sporulation and maintenance were performed as previously described^8,11^.

### Extraction and isolation of the antifungal compounds from *T. palmate*

**Figure 4:**
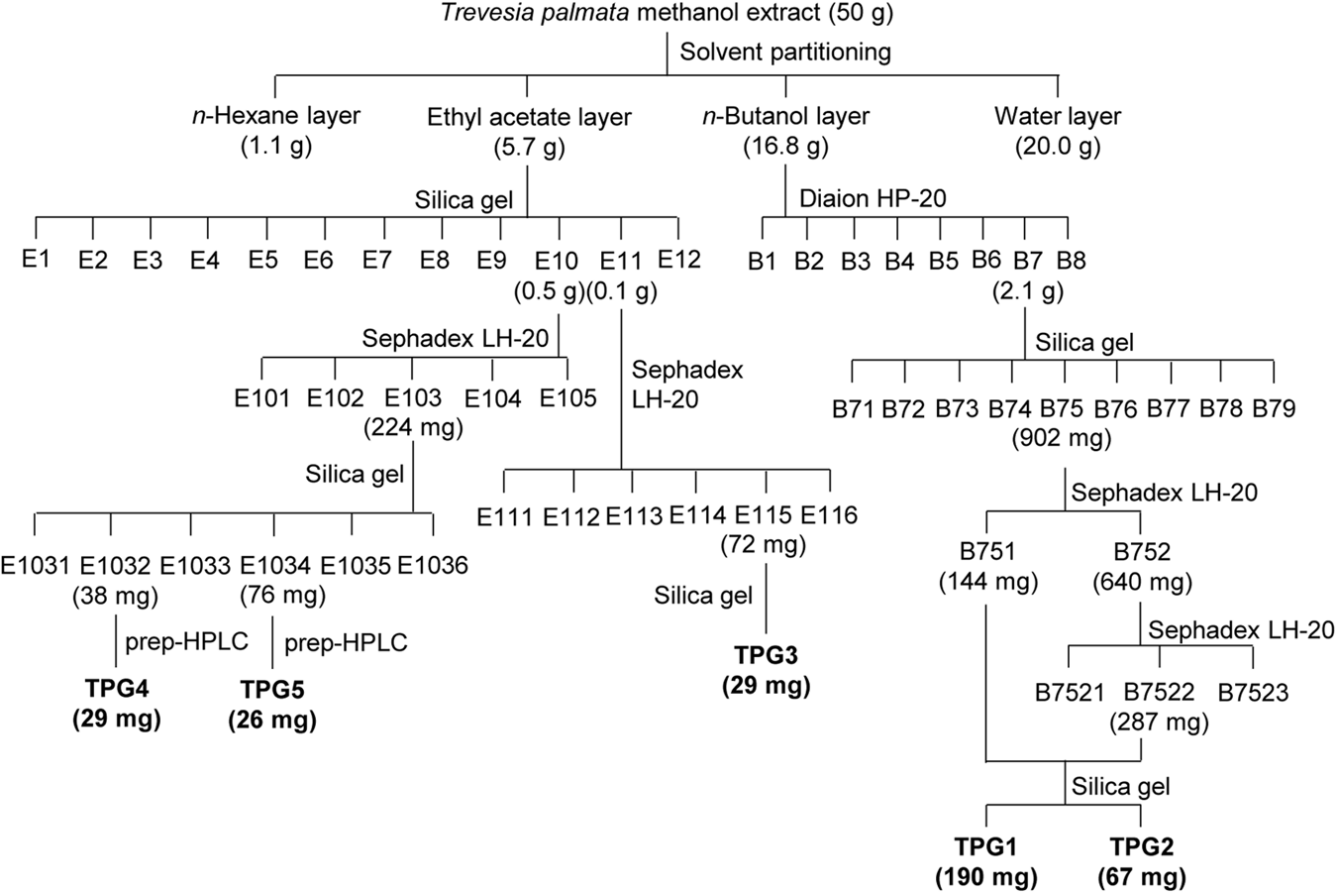
Isolation scheme for the triterpene glycosides from *Trevesia palmata*.

A procedure for the extraction and isolation of the active compounds is shown in a flowchart (Fig. 4). The powder material of *T. palmata* (520 g) was extracted three times with methanol under reflux for 3 h. The extracts were filtered through Whatman No. 1 filter paper, and then, the filtrates were concentrated by a rotary evaporator, giving 50 g of a methanol extract. The whole methanol extract was suspended in 500 ml of water and successively partitioned twice with the same amount of *n*-hexane, ethyl acetate, and *n*-butanol.

The *n*-butanol layer (16.8 g) was subjected to a Diaion HP-20 column and eluted with methanol/water (0:100, 20:80, 40:60, 60:40, 80:20, and 100:0, v/v), yielding seven fractions B1–B7. Fraction B7 (2.1 g), which showed an strong antifungal activity against *M. oryzae*, was loaded onto a silica gel column (230–400 mesh; Merck, Darmstadt, Germany) and successively eluted with a gradient of chloroform/methanol/water (7:3:0, 14:6:1, and 0:20:1, v/v/v), yielding nine fractions B71–B79. The active fraction B75 (902 mg) was successfully separated on a Sephadex LH-20 column (Merck) using chloroform/methanol (1:1, v/v) to yield 2 fractions B751–B752. B752 (640 mg) was successfully separated on a Sephadex LH-20 column with an elution of methanol yielding three fractions B7521–B7523. The active fractions B751 (144 mg) and B7522 (287 mg) were combined and further purified on a silica gel column by using chloroform/methanol/water (14:6:1, v/v/v), and yielded two pure compounds TPG1 (190 mg) and TPG2 (67 mg).

The ethyl acetate layer (5.7 g) was separated by a silica gel column with chloroform/methanol/water (9:1:0, 30:9:1, 14:6:1, 12:8:1, and 0:20:1, v/v/v) to give twelve fractions E01–E12. Antifungal fraction E11 was subjected to Sephadex LH-20 column chromatography to give six fractions E111–E116. TPG3 (29 mg) was purified from E115 fraction (72 mg) by a silica gel column eluted with chloroform/methanol/water (14:6:1, v/v/v). Another antifungal fraction E10 was subjected to Sephadex LH-20 column chromatography to give five fractions E101–E105. E103 fraction (224 mg) was separated by a silica gel column with chloroform/methanol/water (14:6:1, v/v/v) to give six fractions E1031–E1036. TPG4 (29 mg) and TPG5 (26 mg) were finally purified from E1032 (38 mg) and E1034 (76 mg) using a LC-6AD HPLC system (Shimadzu, Kyoto, Japan) equipped with a Polaris C18-A column (21.2 × 250 mm, 10 μm; Agilent, Santa Clara, CA). The column was eluted with a linear gradient (80–100% for 50 min) of aqueous methanol at a flow rate of 5 ml/min. The effluent was monitored with the SPD-M10Avp photodiode array detector (Shimadzu).

### Identification of active compounds

Attenuated total reflection infrared (ATR-IR) spectra were measured on a Smiths IdentifyIR spectrometer (Danbury, CT). Mass spectra were recorded with a single-quadrupole mass spectrometer (Acquity QDa; Waters, Manchester, UK), and HRMS was measured by a Synapt G2 mass spectrometer (Waters). NMR experiments were carried out on Bruker Avance 500 and 700 MHz spectrometers (Burker BioSpin, Rheinstetten, Germany) in methanol-*d*_4_ (99.8 atom% D; Cambridge Isotope Laboratories, Tewksbury, MA). Chemical shifts were referenced to the solvent peaks (*δ*_H_ 3.31 and *δ*_C_ 49.0 for methanol-*d*_4_).

### Acid hydrolysis and sugar analysis

The absolute configuration of the sugar moieties in the structures was determined by the method of Tanaka *et al*.^40^. Each compound (3 mg) was dissolved in 5 ml of 1 M hydrochloric acid and then heated at 90 °C in a water bath for 6 h. The resulting product was dissolved in water after evaporation. The mixture was extracted with ethyl acetate three times. The aqueous layer containing monosaccharides was concentrated to dryness, which was then mixed with 5 mg of _L_-cysteine methyl ester hydrochloride. Anhydrous pyridine (5 ml) was added to the mixture, and then heated at 60 °C for 1 h. The resulting product was mixed with 2 mg of isothiocyanate and further heated at 60 °C for 1 h. The final product was directly analyzed by a Waters 515 HPLC system (Miliford, MA) equipped with a reversed-phase C18 Pursuit XRs column (4.6 × 250 mm, 5 μm; Agilent, Santa Clara, CA). Analysis was performed at 30 °C with a flow rate of 0.8 ml/min, and the elution was carried out using a 25% aqueous acetonitrile containing 0.08% formic acid. Peaks were detected at 250 nm and identified by comparison of the retention time with standards. The retention time of _D_-glucose, _L_-arabinose, and _L_-rhamnose were 16.7, 18.8, and 27.5 min, respectively.

*Compound TPG1*: amorphous powder; [α]_D_^20^ −8.26 (c 0.1, MeOH); IR ν_max_ 3378, 2938, 1692, 1636, 1456, 1385, 1261, 1056, 702 cm^−1^; ^1^H- and ^13^C-NMR, see Tables 2 and 3; MALDI-MS m/z 1081 [M + Na]^+^; MALDIMS/MS m/z 935 [M + Na − 146]^+^, m/z 773 [M + Na − 146 − 162]^+^, 627 [M + Na − 146 − 162 − 146]^+^; HR-ESI-MS m/z 1081.5536 [M + Na]^+^ (Calc. m/z 1081.5559).

*Compound TPG2*: amorphous powder; ^1^H- and ^13^C-NMR, see Tables 2 and 3; MALDI-MS m/z 935 [M + Na]^+^; MALDIMS/MS m/z 773 [M + Na − 162]^+^, 627 [M + Na − 162 − 146]^+^.

*Compound TPG3*: amorphous powder; ^1^H- and ^13^C-NMR, see Tables 2 and 3; MALDI-MS m/z 773 [M + Na]^+^; MALDIMS/MS m/z 627 [M + Na − 146]^+^.

*Compound TPG4*: amorphous powder; ^1^H- and ^13^C-NMR, see Tables 2 and 3; ESI-MS m/z 805 [M + Na]^+^.

*Compound TPG5*: amorphous powder; ^1^H- and ^13^C-NMR, see Tables 2 and 3; ESI-MS m/z 643 [M + Na]^+^; HR-ESI-MS m/z 643.3820 [M + Na]^+^ (Calc. m/z 643.3822).

### *In vitro* antifungal assay

The MIC and IC_50_ values of the isolated compounds against plant pathogenic fungi were determined by the broth microdilution assay using two-fold serial dilutions starting with 256 μg/ml as described by the modified CLSI M38-A method^41^. Briefly, a spore suspension (1 × 10^5^ spores/ml) of phytopathogenic fungi in PDB medium (100 μl) was added to the wells of a 96-well microtiter plate. The stock solutions of the TPGs (25 mg/ml) were diluted 100-fold in the first wells and then, two-fold serial dilutions were performed in PDB medium. The final concentrations of the TPGs were in the range 7.8–256 μg/ml. The inhibitory effects on the fungal growth were determined by the optical density (OD_600_) after incubation for 1–3 days. Growth inhibition (%) was calculated as [1 − (OD_600_ of treatment/OD_600_ of control)] × 100, and the MIC and IC_50_ values were calculated from the concentration–response curves^42^. The assay was performed two times with three replicates for each compound at all concentrations investigated.

### *In vivo* antifungal assay

To evaluate the plant disease control efficacies, the concentration of the solvent extracts (2,000 μg/ml) and TPG1 (125, 250 and 500 μg/ml) were adjusted by dissolving in a 5% methanol solution containing 0.025% Tween 20 solution. The final concentration of the methanol in each treatment did not exceed 5% of the volume. Chemical fungicides (blasticidin S, validamycin, fludioxonil, dimethomorph, flusilazole, and dithianon) and 5% methanol were used as positive and negative controls, respectively. As hosts for the pathogens, tomato (*Solanum lycopersicum* cv. Seokwang), wheat (*Triticum aestivum* cv. Eunpa), barley (*Hordeum sativum* cv. Dongbori), and pepper (*Capsicum annuum* cv. Bugang) were used, which were grown in a greenhouse at 25 ± 5 °C for 1–5 weeks. After treatment with the plant extracts or TPGs, the plants were inoculated with a fungal pathogen and incubated as previously described^42,43^. The experiment was conducted twice with three replicates for each treatment, and the disease control efficacy was calculated with the following equation: control efficacy (%) = 100 × [1 − B/A], where A is the mean lesion area (%) on the leaves or sheaths of the control plants, and B is the mean lesion area (%) on the leaves or sheaths of the treated plants^11,42,43^.

### Statistical analyses

All experiments were performed in triplicate with two trials. The results represented as the means ± standard deviations. Statistical analyses were performed by one-way ANOVA followed by Tukey’s tests using the R-software (version 3.5.0).

## Electronic supplementary material

Supplementary information

## References

[1] Oerke EC (2006). Crop losses to pests. J. Agric. Sci..

[2] Hüter OF (2011). Use of natural products in the crop protection industry. Phytochem. Rev..

[3] Centrell CL, Dayan FE, Duke SO (2012). Natural products as sources for new pesticides. J. Nat. Prod..

[4] Dayan FE, Cantrell CL, Duke SO (2009). Natural products in crop protection. Bioorganic Med. Chem..

[5] Vu TT (2016). *In vitro* antibacterial activity of selected medicinal plants traditionally used in Vietnam against human pathogenic bacteria. BMC Compl. Alternat. Med..

[6] Copping LG, Duke SO (2007). Natural products that have been used commercially as crop protection agents. Pest Manag. Sci..

[7] Monsálvez M (2010). Antifungal effects of *n*-hexane extract and essential oil of *Drimys winteri* bark against Take-All disease. Ind. Crop. Prod..

[8] Choi GJ (2004). Effects of chrysophanol, parietin, and nepodin of *Rumex crispus* on barley and cucumber powdery mildews. Crop Prot..

[9] Devaiah SP, Mahadevappa GH, Shetty HS (2009). Induction of systemic resistance in pearl millet (*Pennisetum glaucum*) against downy mildew (*Sclerospora graminicola*) by *Datura metel* extract. Crop Prot..

[10] Castillo, F., Hernández, D., Gallegos, G., Rodríguez, R. & Aguilar, C. N. Antifungal properties of bioactive compounds from plants in *Fungicides for plant and animal diseases* (eds. Dhanasekaren, D., Thajuddin, N. & Panneerselvam A.) 82–106 (InTech, Rijeka, 2012).

[11] Choi NH (2017). Antifungal activity of sterol and dipsacus saponins isolated from *Dipsacus asper* root against phytopathogenic fungi. Pest. Biochem. Physiol..

[12] Teshima Y (2013). Identification and biological activity of antifungal saponins from shallot (*Allium cepa* L. aggregatum group). J. Agric. Food Chem..

[13] Augustin JM, Kuzina V, Andersen SB, Bak S (2011). Molecular activities, biosynthesis and evolution of triterpenoid saponins. Phytochemistry.

[14] Keukens EAJ (1995). Molecular basis of glycoalkaloid induced membrane disruption. Biochim. Biophys. Acta..

[15] Njateng (2015). Antifungal properties of a new terpernoid saponin and other compounds from the stem bark of *Polyscias fulva* Hiern (Araliaceae). BMC Complement. Altern. Med..

[16] Jebb MHP (1998). A revision of the genus *Trevesia* (Araliaceae). Glasra.

[17] Tommasi ND (2000). Antiproliferative triterpene saponins from *Trevesia palmata*. J. Nat. Prod..

[18] Rahman KMH (2014). Phytochemical screening, antihypergycemic and analgesic activity studies with methanol extract of *Trevesia palmata* leaves. World J. Pharm..

[19] Şenel G, Gülcemal D, Masullo M, Piacente S, Karayildirim T (2014). Oleanane-type glycosides from *Tremastelma palaestinum* (L.) janchen. Chem. Biodivers..

[20] Saito S (1990). Saponins from the leaves of *Aralia elata* SEEM. (Araliaceae). Chem. Pharm. Bull..

[21] Kayce P, Kirmizigul S (2017). Isolation and identification of a new saponin from *Cephalaria aytachii*. Nat. Prod. Res..

[22] Aguirre CM (2006). Topical anti-inflammatory activity of 2a-hydroxy pentacyclic triterpene acids from the leaves of *Ugni molinae*. BioorganicMed. Chem..

[23] Nishimura K, Miyase T, Noguchi H (1999). Triterpenoid saponins from *Ilex kudincha*. J. Nat. Prod..

[24] Bang SC (2005). Antitumor activity of *Pulsatilla koreana* saponins and their structure–activity relationship. Chem. Pharm. Bull..

[25] Hung TM (2005). Cytotoxic Saponins from the Root of *Dipsacus asper* Wall. Arch. Pharm. Res..

[26] Li W (2013). Isolation of nematicidal triterpenoid saponins from *Pulsatilla koreana* root and their activities against *Meloidogyne incognita*. Molecules.

[27] Li W (2014). Anti-inflammatory and PPAR transactivational effects of oleanane-type triterpenoid saponins from the roots of *Pulsatilla koreana*. Biomol. Ther..

[28] Saito S (1993). Comparison of cytoprotective effects of saponins isolated from leaves of *Aralia elata* SEEM. (Araliaceae) with synthesized bisdesmosides of oleanoic acid and hederagenin on carbon tetrachloride-induced hepatic injury. Chem.Pharm Bull..

[29] Sarıkahya NB, Kırmızıgül S (2012). Antimicrobially active hederagenin glycosides from *Cephalaria elmaliensis*. Planta Med..

[30] Sparg SG, Light ME, van Staden J (2004). Biological activities and distribution of plant saponins. J. Ethnopharmacol..

[31] Abbruscato P (2014). Triterpenoid glycosides from *Medicago sativa* as antifungal agents against *Pyricularia oryzae*. J. Agric. Food Chem..

[32] Yoon MY (2011). Potent *in vivo* antifungal activity against powdery mildews of pregnane glycosides from the roots of *Cynanchum wilfordii*. J. Agric. Food Chem..

[33] Shi W, Liu HW, Guo X, Hou L, Gao JM (2016). Triterpenoids from the stems of *Schisandra grandiflora* and their biological activity. J. Asian Nat. Prod. Res..

[34] Lahlou EIH, Hirai N, Kamo T, Tsuda M, Ohigashi H (2001). Actinidic acid, a new triterpene phytoalexin from unripe kiwi fruit. Biosci. Biotechnol. Biochem..

[35] Njateng GSS (2015). Antifungal properties of a new terpernoid saponin and other compounds from the stem bark of *Polyscias fulva* Hiern (Araliaceae). BMC Complement. Altern. Med..

[36] Konishi M (1998). Triterpenoid saponins from *Hedyotis nudicaulis*. Phytochemistry.

[37] Morrissey JP, Osbourn AE (1999). Fungal resistance to plant antibiotics as a mechanism of pathogenesis. Microbiol. Mol. Biol. Rev..

[38] Ito S, Nagata A, Kai T, Takahara H, Tanaka S (2005). Symptomless infection of tomato plants by tomatinase producing *Fusarium oxysporum* formae speciales nonpathogenic on tomato plants. Physiol. Mol. Plant Pathol..

[39] Berniyanti T, Mahmiyah E (2015). Microbiological studies on the production of antimicrobial agent by saponin Aloe vera Linn against *Streptococcus sanguinis*. Res. J. Microbiol..

[40] Tanaka T, Nakashima T, Ueda T, Tomii K, Kouno I (2007). Facile discrimination of aldose enantiomers by reversed-phase HPLC. Chem. Pharm. Bull..

[41] Espinel-Ingroff A (2005). Quality control and reference guidelines for CLSI broth microdilution susceptibility method (M38-A document) for amphotericin B, itraconazole, posaconazole, and voriconazole. J. Clin. Microbiol..

[42] Han JW (2018). *In vivo* disease control efficacy of isoquinoline alkaloids isolated from *Corydalis ternata* against wheat leaf rust and pepper anthracnose. J. Microbiol. Biotechnol..

[43] Han JW (2018). *In vivo* assessment of plant extracts for control of plant diseases: A sesquiterpene ketolactone isolated from *Curcuma zedoaria* suppresses wheat leaf rust. *J. Environ. Sci. Health*. Part B.

