# Supplementary material for: Identification of novel compounds, oleanane- and ursane-type triterpene glycosides, from Trevesia palmata: their biocontrol activity against phytopathogenic fungi
Source: Sci Rep. 2018 Sep 28;8:14522. doi: 10.1038/s41598-018-32956-4 (PMC6162204; doi:10.1038/s41598-018-32956-4)
Supplement: Supplementary file 1 — Supplementary information [file 41598_2018_32956_MOESM1_ESM.pdf]

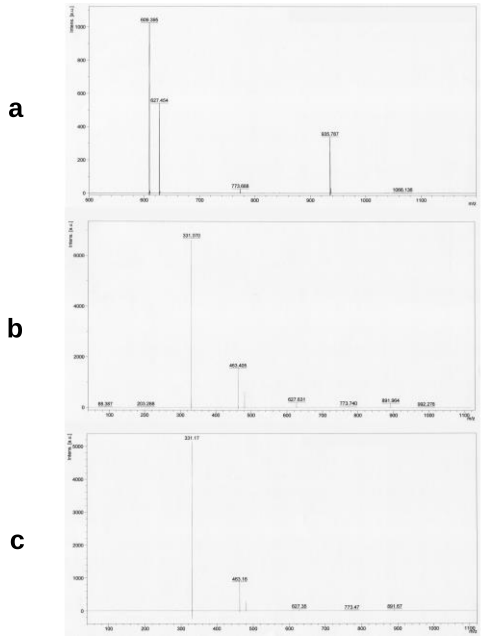

Supplementary Fig. S1. . MALDI-MS/MS fragmentation of m/z 1081, 935, and 773 $[M + Na]^+$ for compounds TPG1 (a), TPG2 (b), and TPG3 (c) isolated from *Trevesia palmata*.

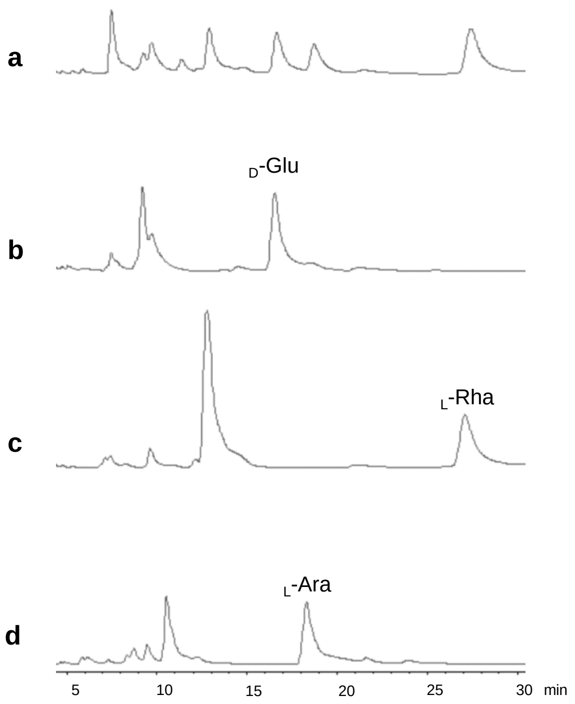

Supplementary Fig. S2. HPLC chromatograms for the derivatives of compound TPG1 hydolysate (a) and standard sugars (b–d).

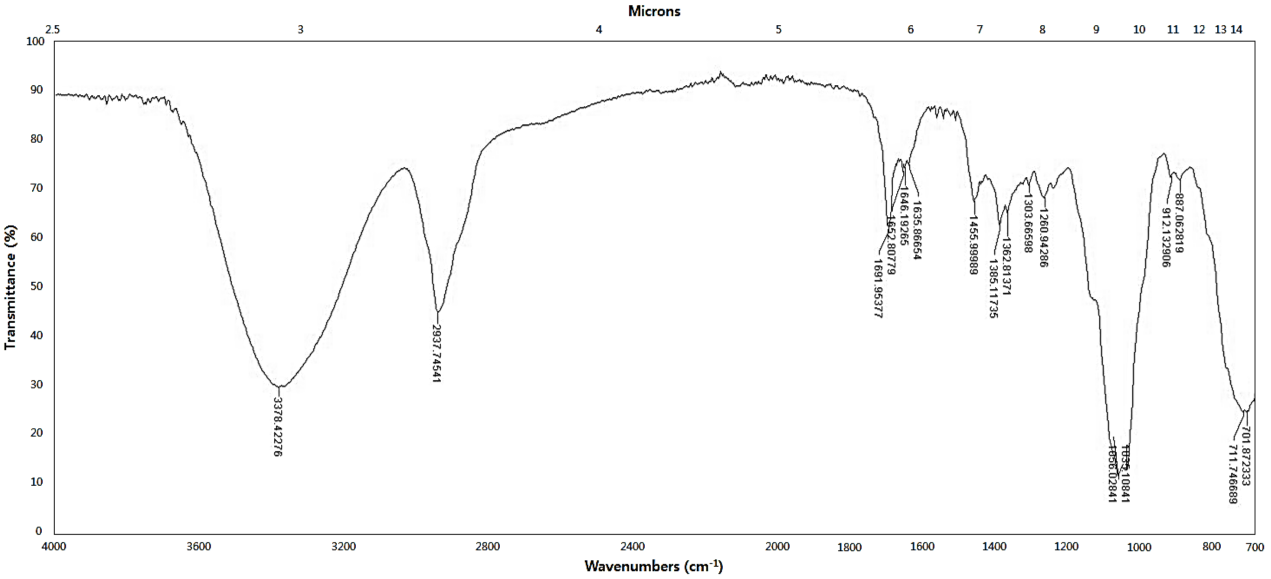

Supplementary Fig. S3. IR spectrum of compound TPG1 isolated from *Trevesia palmata*.

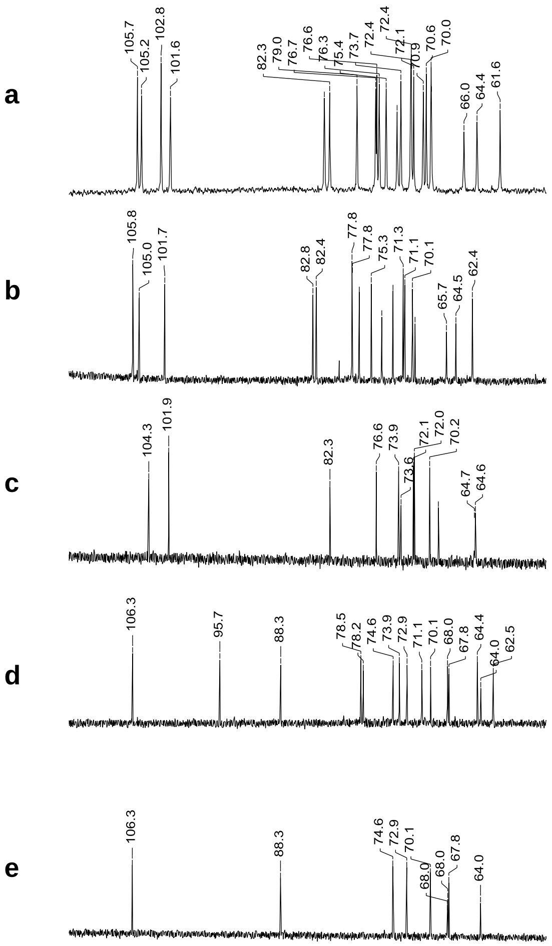

Supplementary Fig. S4. Comparison of ^{13}C-NMR spectra of oligosaccharide moiety of triterpene glycosides TPG1 (a), TPG2 (b), TPG3 (c), TPG4 (d), and TPG5 (e).

Pathogen	IC_{50} (μg/ml)				
	TPG1	TPG2	TPG3	TPG4	TPG5
Agrobacterium tumefaciens	> 256	> 256	> 256	> 256	>256
Burkholderia glumae	> 256	> 256	> 256	> 256	>256
Clavibacter michiganensis subsp. *michiganensis*	158	195	78	> 256	112
Pectobacterium carotovorum subsp. *carotovorum*	> 256	> 256	> 256	> 256	>256
Pseudomonas syringae pv. *actinidiae*	> 256	> 256	> 256	> 256	>256
Xantomonas arboricola pv. *pruni*	> 256	> 256	> 256	> 256	>256
Ralstonia solanacearum	> 256	> 256	> 256	> 256	>256

Supplementary Table S1. IC_{50} values of TPGs against phytopathogenic bacteria. Half maximal inhibitory concentration (IC_{50}) values of TPGs against plant pathogenic bacteria were determined by broth microdilution assay using two-fold serial dilutions starting with 256 μg/ml as described by the modified CLSI M38-A method. Bacterial suspensions (1×10^4 cells/ml) grown in tryptic soy broth (BD Biosciences) was used as inocula. Aqueous 5% methanol solution without the chemical were also used as a control. The inhibitory effects on the growth of microbes were determined after incubation for 1–3 days. The optical density at 600 nm (OD_{600}) of each well was recorded using a microplate reader. Growth inhibition (%) was calculated as [1 - (OD_{600} of treatment / OD_{600} of control)] × 100. IC_{50} values were calculated from the concentration–response curves.
